# Relationship Between Chemical Structures of Phytochemicals, Synthetic Phytochemical Analogs, and Antibiotics and Their In Vitro Growth-Inhibitory Effects Against Colorectal Cancer-Causing Bacteria

**DOI:** 10.3390/molecules31122151

**Published:** 2026-06-18

**Authors:** Barbora Fiserova, Tomas Kudera, Hana Subrtova-Salmonova, Tereza Navratilova, Ladislav Kokoska

**Affiliations:** 1 Department of Crop Sciences and Agroforestry, Faculty of Tropical AgriSciences, Czech University of Life Sciences Prague, Kamycka 129, 16500 Prague, Czech Republic; fiserovab@ftz.czu.cz (B.F.); kuderat@ftz.czu.cz (T.K.); 2 Department of Microbiology, Nutrition and Dietetics, Faculty of Agrobiology, Food and Natural Resources, Czech University of Life Sciences Prague, Kamycka 129, 16500 Prague, Czech Republic; salmonova@af.czu.cz; 3 Department of Physical Chemistry, Faculty of Chemical Engineering, University of Chemistry and Technology, Technická 5, 16628 Prague, Czech Republic; navratit@vscht.cz

**Keywords:** intestinal cancer, oncogenic bacteria, structure–activity relationship, phytochemicals, 8-hydroxyquinoline derivatives, antibacterial activity, gut microbiota, SAR

## Abstract

Colorectal cancer (CRC) has been increasingly associated with gut microbiota dysbiosis and the presence of specific bacterial pathogens. This study evaluated the in vitro growth-inhibitory activity of 18 biologically active compounds, including phytochemicals, synthetic analogs, and clinically used antibiotics, against CRC-associated bacterial strains. Minimum inhibitory concentrations (MICs) were determined using the broth microdilution method and analyzed in relation to chemical structure. Conventional antibiotics, particularly tetracycline and ciprofloxacin, exhibited the strongest antibacterial activity. Among non-antibiotic compounds, nitroxoline and carbadox showed moderate activity, whereas quaternary benzylisoquinoline-derived alkaloids and polyphenols were less effective. Structure–activity relationship analysis suggested that aromatic heterocyclic scaffolds, electron-withdrawing substituents, and metal-chelating groups contribute to antibacterial potency. We obtained novel MIC data for several compounds, including ferron and oxyquinoline, against underexplored CRC-associated bacterial strains. These findings expand current knowledge of the antibacterial activity of structurally diverse compounds against CRC-associated bacteria and provide a basis for future studies on microbiota-targeted antimicrobial strategies.

## 1. Introduction

Colorectal cancer (CRC), defined as an adenocarcinoma of the large intestine, caused 0.9 million deaths worldwide in 2022 [1]. Various therapeutic modalities, including surgery, radiotherapy, and chemotherapy, are available for CRC; however, the damage to normal cells caused by such treatments may cause serious adverse effects [2]. Gut dysbiosis, characterized by an imbalance in the composition and metabolic activity of intestinal microbiota and often accompanied by digestive disturbances such as diarrhea, is considered one of the major factors associated with an increased risk of CRC development [3,4]. In a healthy intestinal environment, the gut microbiota maintains host homeostasis through multiple mechanisms, including nutrient metabolism, maintenance of the intestinal barrier, modulation of immune responses, and protection against pathogenic microorganisms [5,6]. However, disruption of this balanced microbial community can lead to the overgrowth of opportunistic and potentially pathogenic bacterial strains, while beneficial commensal bacteria decline. Such alterations may result in impaired intestinal barrier function, increased intestinal permeability, and chronic low-grade inflammation, all of which contribute to a microenvironment favorable for carcinogenesis [7,8].

Several microbial species associated with dysbiosis have been implicated in CRC pathogenesis. These microorganisms can promote tumor development through the production of genotoxins, virulence factors, and carcinogenic metabolites, as well as by inducing oxidative stress and inflammatory signaling pathways in the colonic epithelium. Furthermore, dysbiotic microbiota may alter host metabolic processes, including bile acid transformation and short-chain fatty acid production, thereby affecting epithelial cell proliferation, apoptosis, and DNA stability. The resulting inflammatory and mutagenic environment can promote genomic instability and facilitate the initiation and progression of colorectal tumors. Consequently, the relationship between gut microbial imbalance and CRC development has become a key area of research, highlighting the significance of microbiota-targeted preventive and therapeutic strategies [9].

Development of CRC is accompanied by a progressive shift in gut microbiota composition at the phylum level, characterized by depletion of protective *Bacillota* (formerly *Firmicutes*) and enrichment of pro-inflammatory and genotoxic taxa from *Bacteroidota*, *Pseudomonadota*/*Proteobacteria*, and *Fusobacteriota*. The loss of *Bacillota*, particularly butyrate-producing members such as *Faecalibacterium*, *Roseburia*, and *Lachnospiraceae*, reduces short-chain fatty acid production, impairing epithelial barrier integrity and anti-inflammatory signaling, thereby facilitating tumor initiation [10]. Elevated levels of several invasive bacterial strains, such as *Bacteroides fragilis*, *Clostridium septicum*, *Escherichia coli*, *Fusobacterium necrophorum*, *F. nucleatum*, *Peptostreptococcus anaerobius*, and *Streptococcus bovis*, have been reported in the stool of patients with CRC [11,12]. In contrast, specific members of *Bacteroidota*, notably enterotoxigenic *B. fragilis*, contribute to early carcinogenesis through secretion of *B. fragilis* toxin, which disrupts E-cadherin and activates β-catenin and NF-κB signaling pathways, thereby promoting chronic inflammation. Expansion of Proteobacteria, particularly *E. coli* harboring the polyketide synthase genomic island, further drives tumor progression via production of the genotoxin colibactin, which induces DNA damage and mutational signatures in host cells. At later stages, *Fusobacteriota* (especially *F. nucleatum*) become enriched within tumor tissue, where they promote tumor growth through adhesion-mediated activation of β-catenin signaling (via FadA), modulation of immune responses, and stimulation of pro-inflammatory pathways such as TLR4/MYD88/NF-κB [13]. This dynamic microbial evolution aligns with the driver–passenger model, in which early “driver” bacteria initiate tumorigenic processes, while later “passenger” species exploit the tumor microenvironment and further promote disease progression [14]. Collectively, these shifts illustrate how phylum-level dysbiosis reflects the underlying functional changes, the loss of metabolic homeostasis, and the gain of inflammatory and genotoxic activities, which contribute to CRC initiation and progression.

Although epidemiological studies suggest that long-term antibiotic exposure may contribute to CRC development through disruption of gut microbiota, targeting of CRC-associated pathogenic bacteria has emerged as a potential therapeutic strategy. Therefore, the development of antibacterial agents that specifically inhibit tumor-promoting gut microbes may represent a promising approach for CRC prevention or adjunctive treatment. Studies that do support the CRC association show that antibiotic exposure may increase colon cancer risk, likely through disruption of gut microbiota. For example, epidemiological research reported that antibiotic use was associated with a higher risk of colon cancer (adjusted OR ≈1.49 in individuals <50 years) [15]. Oral antibiotic use has been associated with an increased risk of colon cancer, highlighting the potential role of antibiotic-induced microbiome alterations in colorectal carcinogenesis [16]. The association between chemical structure and biological activity represents a fundamental principle in antimicrobial research and drug discovery, supporting the rational selection and optimization of bioactive compounds. Structure–activity relationship (SAR) analysis is therefore a valuable approach for identifying promising structural candidates. The analysis of such relationship enables the determination of the chemical moieties responsible for evoking the targeted biological effect in the organism [17]. In recent years, increasing attention has been directed toward natural phytochemicals and their synthetic analogs as promising antimicrobial agents, particularly in the context of rising antibiotic resistance and the need for more microbiota-targeting strategies. As highlighted by Kudera et al.’s review [18], plant-derived quaternary benzylisoquinoline-derived alkaloids and related nitrogen-containing heterocyclic compounds exhibit a wide spectrum of biological activities, with antimicrobial effects strongly influenced by their structural features, including substitution patterns, electronic properties, and metal-chelating capacity. These compounds often act through diverse mechanisms, such as disruption of metal homeostasis, redox cycling, and interference with essential cellular processes, which can result in inhibition of specific microbial taxa [19].

This structural diversity is particularly relevant in the context of CRC, where gut microbiota dysbiosis plays a key role in disease initiation and progression. CRC-associated bacterial strains, including *F. nucleatum*, *B. fragilis*, and *P. anaerobius*, contribute to carcinogenesis through mechanisms such as toxin production, chronic inflammation, and modulation of host signaling pathways. In contrast to conventional broad-spectrum antibiotics, several phytochemicals and their synthetic derivatives have been reported to exhibit distinct antibacterial activity profiles and mechanisms of action, including metal chelation, redox modulation, and interference with bacterial metabolism. These properties make them attractive candidates for future investigation as microbiota-directed antimicrobial agents. However, their selectivity toward CRC-associated bacteria relative to beneficial commensal microorganisms remains to be established [20].

In this context, both natural compounds and their synthetic analogs represent a valuable platform for exploring SAR and identifying key chemical moieties responsible for antimicrobial efficacy. While clinically used antibiotics provide essential reference points with well-characterized activity profiles, structurally diverse phytochemicals offer complementary mechanisms that may be particularly suitable for microbiota-directed interventions [20]. Therefore, this study aims to investigate the relationship between chemical structure and in vitro growth-inhibitory activity of selected phytochemicals, their synthetic analogs, and conventional antibiotics against CRC-associated bacteria, with the goal of identifying structural determinants underlying antimicrobial effects and their potential relevance for CRC-associated microbiota modulation.

The compounds included in this study were selected to represent several structurally distinct classes of biologically active molecules with documented antimicrobial activity and potential relevance to intestinal microbiota modulation [18]. These comprised naturally occurring phytochemicals (polyphenols and alkaloids) and synthetic analogs of plant-derived compounds (quinolines, quinoxaline derivatives, and pyrithione complexes), with clinically used antibiotics serving as reference compounds. Particular emphasis was placed on nitrogen-containing heterocyclic molecules, such as 8-hydroxyquinoline and quinoxaline derivatives, because of their reported antimicrobial effects, metal-chelating properties, and structural suitability for SAR analysis [21,22,23]. In addition, several compounds, including ferron, oxyquinoline, and chloroxine, were selected because information regarding their activity against CRC-associated bacteria is currently limited or unavailable. For example, chloroxine has been used as an intestinal antiseptic, whereas tannin-containing preparations, such as tannalbin, have traditionally been employed as antidiarrheal agents [24]. Collectively, the selected compounds provide a chemically diverse dataset suitable for comparing antibacterial activity patterns and exploring potential relationships between molecular structure and growth-inhibitory effects against CRC-associated bacterial strains. Although the majority of these compounds were included in our previous study, which preliminarily investigated their selective antimicrobial potential against diarrheagenic bacteria and probiotic intestinal strains, as well as their antiproliferative potential [20], the susceptibility of CRC-associated bacteria to these classes of compounds has not yet been determined and the associated SAR is not fully understood. Therefore, we decided to compare in vitro growth-inhibitory effects of various biologically active compounds, such as 8-hydroxyquinolines, quinoxalines, polyphenols and phenolic compounds, and plant-derived quaternary benzylisoquinoline-derived alkaloids, together with commercially available antibiotics used for treatment of diarrhea against CRC-associated bacterial strains, using broth microdilution method to determine the relationship between their chemical structures and antibacterial activities. Antibacterial activity was expressed as the minimum inhibitory concentration (MIC) for each strain, and the arithmetic mean MIC ($\bar{x}$MIC) was calculated to represent the overall actions against the tested CRC-associated bacterial strains. 

## 2. Results

In general, conventional antibiotics exhibited broader and stronger activity (MICs ≥ 0.0625 μg/mL) compared to phytochemicals and their synthetic analogs (MICs ≥ 0.5 μg/mL). Although ciprofloxacin, a representative of quinoline-based antibiotics, produced lowest MIC value among all compounds tested (MIC = 0.0625 μg/mL against *E. coli*) and had generally good antibacterial activity ($\bar{x}$MIC = 1.7 μg/mL), tetracycline demonstrated the most consistent growth-inhibitory effect against CRC-associated pathogens, with MICs ranging from 0.125 to 2 μg/mL ($\bar{x}$MIC = 1.1 μg/mL). Quinoline, nitroxoline, quinoxaline-di-N-oxide, and carbadox produced strong to moderate effects, with MICs ranging from 0.5 to 32 μg/mL ($\bar{x}$MIC ranging from 10.9 to 11.0 μg/mL). Similarly, chloramphenicol and vancomycin showed strong to moderate activity against all tested bacterial strains except *E. coli* (MIC > 32 μg/mL), with MICs ranging from 1 to 16 μg/mL ($\bar{x}$MIC = 15.6 μg/mL) and from 1 to 32 μg/mL ($\bar{x}$MIC = 19.0 μg/mL), respectively. Ceftriaxone ($\bar{x}$MIC = 16.5 μg/mL) and metronidazole ($\bar{x}$MIC = 18.8 μg/mL) were effective against most bacteria except *B. fragilis* (resistant to ceftriaxone), *C. septicum*, and *S. bovis* (resistant to metronidazole). Coordination complexes of pyrithione and zinc produced moderate effects against all bacterial strains tested, with MICs ranging from 4 to 32 μg/mL. Berberine chloride, bismuth subsalicylate, chloroxine, ferron, olaquindox, oxyquinoline, salicylic acid, sanguinarine chloride, and tannic acid exhibited weak or no antibacterial activity ($\bar{x}$MIC = 30.3–950.9 μg/mL). Observed MIC values and calculated $\bar{x}$MICs of phytochemicals, their synthetic analogs, and antibiotics are shown in Table 1. *S. bovis*, *B. fragilis*, and *E. coli* were the most susceptible bacteria overall. Solvent controls (DMSO and 96% ethanol at 1% *v*/*v*) did not inhibit bacterial growth of any tested strain, confirming that observed MIC values reflect compound-specific activity.

**Figure 1:**
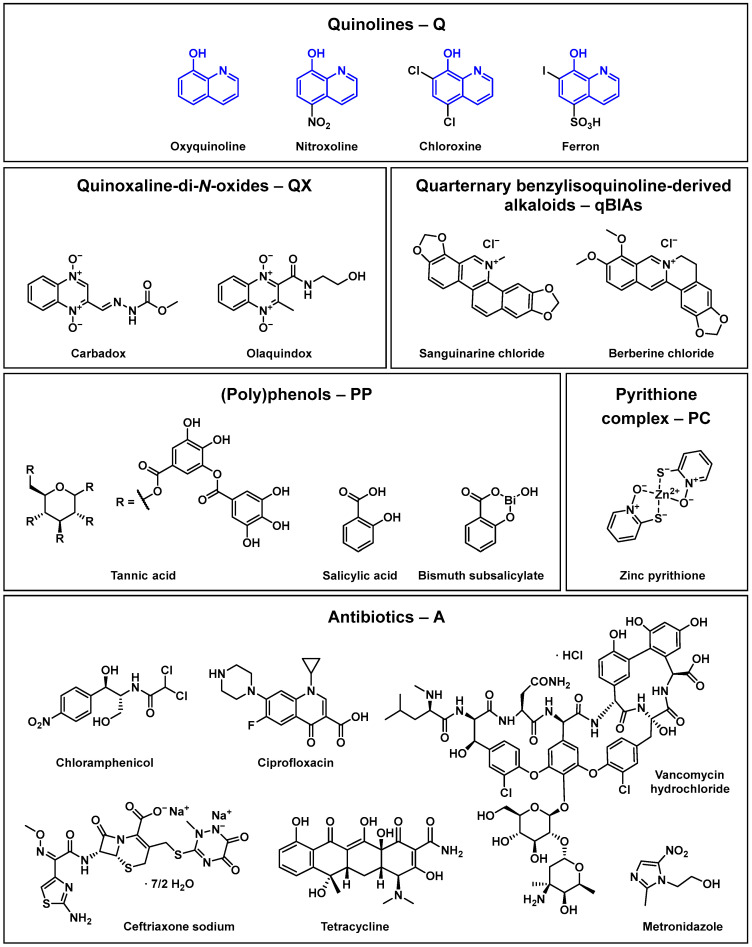
Molecular structures of studied compounds. Blue color highlights the 8-hydroxyquinoline moiety characteristic of the corresponding compounds.

In summary, representatives of tetracycline, fluoroquinolone, amphenicol, cephalosporin, nitroimidazole, and glycopeptide antibiotics were the most active group of antimicrobial agents assayed in this study ($\bar{x}$MIC = 1.1–19.0 μg/mL), followed by quinolines ($\bar{x}$MIC = 10.9–384.0 μg/mL), quinoxaline derivatives ($\bar{x}$MIC = 11.1–112.0 μg/mL), and quaternary benzylisoquinoline-derived alkaloids ($\bar{x}$MIC = 43.4–914.3 μg/mL). Molecules based on the structure comprising a benzene ring fused to a pyridine or pyrazine were among some of the most active substances present in all groups of agents producing significant effect against CRC bacteria tested, whereas many of them shared quinoline moiety (Figure 1). Polyphenols were the least active group, with $\bar{x}$MIC ranging from 768.0 to 950.9 μg/mL.

The linear mixed-effects model converged successfully and included 126 observations from 18 compounds. The model showed strong explanatory power, with marginal R^2^ = 0.655 and conditional R^2^ = 0.880, indicating that the fixed effects explained 65.5% of the variation in MICs, while the full model including compound identity explained 88.0%. Compound class had a significant effect on MICs, F(5, 12) = 8.32, *p* = 0.001, and bacterium also had a significant effect, F(6, 72) = 2.70, *p* = 0.020. The compound class × bacterium interaction was significant, F(30, 72) = 2.38, *p* = 0.001, indicating that the effect of compound class on MICs depended on the bacterial species tested. The random effect of compound was substantial, with ICC = 0.653, supporting the inclusion of compound identity as a random effect. As shown in Table 2, when antibiotics and phytochemicals/synthetic analogs were evaluated together, antibiotics showed the lowest estimated marginal MICs for four of the seven bacterial species: *C. septicum* = 14.77 µg/mL, *F. necrophorum* = 7.63 µg/mL, *F. nucleatum* = 5.94 µg/mL, and *P. anaerobius* = 2.25 µg/mL. Among the phytochemicals and synthetic analogs, the pyrithione complex showed the most promising overall profile, with the lowest estimated marginal MICs for *B. fragilis* = 8.00 µg/mL, *E. coli* = 4.00 µg/mL, and *S. bovis* = 8.00 µg/mL, and low estimated MIC values for several other bacteria. Quinoxaline-di-N-oxides also showed low estimated MIC values for selected bacteria, especially *P. anaerobius* = 8.25 µg/mL. In contrast, quaternary benzylisoquinoline-derived alkaloids and (poly)phenols generally showed higher estimated MIC values, indicating weaker antimicrobial activity.

**Figure 2:**
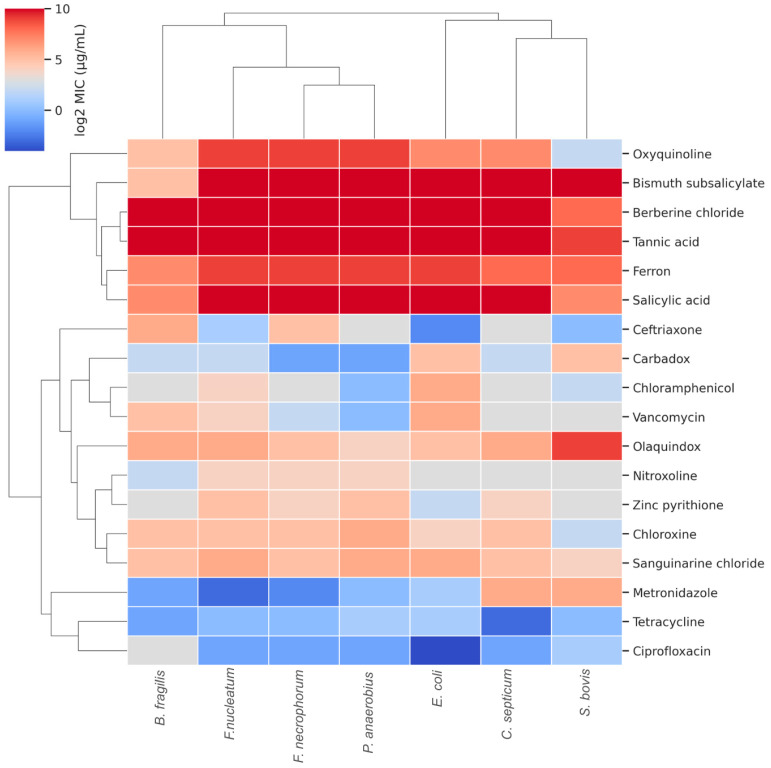
Heatmap with hierarchical clustering of MIC values of tested compounds against CRC-associated bacterial strains.

MIC values were log_2_-transformed prior to clustering. Values exceeding the highest tested concentration (>512 µg/mL) were set to 1024 µg/mL for analysis. The correlation between antimicrobial activities and chemical structure of the tested compounds was further evaluated using hierarchical clustering and heatmap analysis (Figure 2). The clustering revealed distinct groups of compounds with similar antibacterial profiles across CRC-associated bacterial strains. A strong clustering pattern was observed among conventional antibiotics (tetracycline, ciprofloxacin, chloramphenicol, and ceftriaxone), which were grouped together due to their consistently low MIC values, indicating broad-spectrum inhibitory activity. Another distinct cluster included quinoline and quinoxaline derivatives (nitroxoline, chloroxine, oxyquinoline, carbadox, and olaquindox), suggesting similar mechanisms of action and moderate antibacterial activity across multiple strains. Zinc pyrithione clustered closely with quinoline derivatives, which may be attributed to its metal-chelating properties and comparable antimicrobial spectrum. In contrast, phenolic compounds (tannic acid, salicylic acid, and bismuth subsalicylate) together with quaternary benzylisoquinoline-derived alkaloids (berberine chloride and sanguinarine chloride) formed a separate cluster characterized by generally high MIC values, indicating weaker antibacterial activity. Ferron showed partial association with this group, likely due to its high MIC values and limited antibacterial efficacy. Overall, the clustering patterns correspond with the chemical classification of the compounds and highlight similarities in their biological activity profiles against CRC-associated bacterial strains.

## 3. Discussion

The susceptibility of most bacteria tested in this study to antibiotics has previously been documented in the literature, and the MIC values reported align well with our findings [20]. Nevertheless, MICs for ciprofloxacin against *C. septicum* and *F. necrophorum*; chloramphenicol and metronidazole against *C. septicum* and *S. bovis*; vancomycin against *F. necrophorum*; and ceftriaxone against *C. septicum*, *F. necrophorum*, and *P. anaerobius* were determined for the first time. In contrast, MICs of phytochemicals and their synthetic analogs have previously been published only to a limited extent. For example, oxyquinoline has been tested against *B. fragilis* ATCC 25285 and *F. nucleatum* ATCC 25586, with MIC values of 32 and 4 µg/mL, respectively. Reported susceptibility of *B. fragilis* corresponds well with our results, whereas *F. nucleatum* was more sensitive, likely because of the different strain tested [20,25]. In another study, sanguinarine chloride produced MICs in the range of 1–4 µg/mL against various strains of *F. nucleatum*. The slightly higher MIC values observed in our experiment may be attributed to differences in the susceptibility of clinical isolates, as well as minor methodological variations, specifically the use of Mycoplasma broth BBL as the growth medium, compared to the approach used by Dzink and Socransky [26]. In accordance with the literature data [27,28], tannic acid did not produce any significant effect against all bacteria tested. Except for a single study reporting MIC of oxyquinoline against *F. nucleatum* [25], there are no data on the growth-inhibitory effects of phytochemicals and synthetic phytochemical analogs tested against *C. septicum*, *F. necrophorum*, *S. bovis*, and *P. anaerobius*. Moreover, no data on the antibacterial action of ferron against CRC-related bacteria exist in the literature.

Among all the bacteria tested in this study, the susceptibility of *E. coli* to antibiotics, phytochemicals, and their synthetic analogs was the most extensively studied. Although most results describing sensitivity of *E. coli* to antibiotics in the literature correspond well with our findings [29], its resistance to several agents has also been described. For example, the MIC of metronidazole, previously determined using the agar dilution method against clinical isolates of *E. coli* obtained from patients with intra-abdominal infection and abscesses, ranged from 16 to 128 µg/mL [30], which differs from our results. Moreover, in the 2012 study on cefixime, the standard *E. coli* had MIC ~0.75 µg/mL, while clinical isolates had MICs of 8–64 µg/mL [31]. The MIC of ceftriaxone against *E. coli* MG1655 was reported as 0.0625 µg/mL by Ching and Zaman [32]. Additionally, Hansen et al. [31] reported chloramphenicol resistance in *E. coli* strains with MIC values ≥64 µg/mL. In contrast, another study [33] found lower MICs of 6 µg/mL for the *E. coli* strain PHL628. Although the methodology was consistent with ours, the difference in MIC values may be attributed to the use of a different bacterial strain. Among the tested, the MIC of nitroxoline, sanguinarine chloride, and chloroxine against *E. coli* agree with our results [20,34,35,36]. Only two studies address the activity of zinc pyrithione against *E. coli*, with only one providing quantitative results; Dinning et al. [37] reported MIC of 4500 µg/mL for *E. coli* NCIMB 10000, which is 1000 times higher than in our results. Olaquindox showed an initial MIC of 8 µg/mL for *E. coli* ATCC 25922, but resistant strains exposed to sub-MIC levels developed MICs up to 64 µg/mL [38], while Hansen et al. [31] reported MICs ranging from 64 to ≥128 µg/mL in resistant isolates. Finally, McGowan et al. [39] demonstrated that oxyquinoline and its derivatives were inactive against *E. coli*, with MIC values ≥256 µg/mL. This suggests that the 8-hydroxyquinoline derivatives evaluated in this study were not effective inhibitors of *E. coli* growth, which aligns with our observations. There are no previous records of MICs of *E. coli* ATCC 35218 with nitroxoline, carbadox, ceftriaxone, chloroxine, oxyquinoline, zinc pyrithione, metronidazole, and vancomycin. Although *P. anaerobius* and *C. septicum* are the least studied among the examined bacteria, available data indicate that they may exhibit a more resistant or less predictable antibiotic susceptibility profile compared to the more responsive *S. bovis* and *B. fragilis*. These findings emphasize the superior performance of conventional antibiotics while highlighting moderate activity among several synthetic phytochemical analogs. They also underscore the importance of standardized methodology in MIC determination and contribute new antimicrobial benchmarks for underexplored CRC-related pathogens.

Among the quinoline-class compounds, nitroxoline, chloroxine, oxyquinoline, and ferron share the 8-hydroxyquinoline (8-HQ) scaffold, a fused aromatic system bearing a phenolic hydroxyl group and a heterocyclic nitrogen atom that together form a bidentate metal-chelating pharmacophore responsible for their baseline antibacterial activity. However, the nature and position of substituents on this core structure critically modulate biological effects. Nitroxoline (5-nitro-8-hydroxyquinoline) exhibits the strongest activity, which can be attributed to the presence of the nitro group (-NO_2_), a highly electron-withdrawing substituent that significantly decreases electron density within the aromatic system. This effect increases the acidity of the hydroxyl group and enhances metal-binding affinity of the 8-hydroxyquinoline scaffold. In addition, the oxygen atoms of the nitro group may contribute to metal ion coordination, further supporting chelation-driven antimicrobial mechanisms [21,40]. Although nitroxoline and zinc pyrithione demonstrated promising antibacterial activity, their potential application for microbiota-targeted interventions depends on achieving sufficient concentrations within the intestinal lumen. Nitroxoline is rapidly absorbed after oral administration and is primarily used for urinary tract infections, which may limit its direct exposure to colonic microbiota. Consequently, targeted delivery systems, including enteric-coated formulations, pH-responsive encapsulation, or controlled-release carriers, may be required to prolong intestinal residence time and enhance local activity. Similarly, zinc pyrithione exhibits antimicrobial activity through disruption of metal homeostasis, but its intestinal pharmacokinetics remain poorly characterized. Future studies should evaluate formulation approaches capable of improving local intestinal exposure while minimizing systemic effects. In contrast, chloroxine, bearing two chlorine atoms, is the most lipophilic compound in the quinoxaline series, which may enhance membrane permeability and intracellular access. However, the comparatively high lipophilicity of oxyquinoline relative to nitroxoline indicates that lipophilicity alone cannot account for the observed activity differences, and electronic effects associated with the substituents likely also contribute [21,40]. Oxyquinoline, lacking additional substituents, serves as a baseline compound with moderate activity, reflecting the intrinsic chelating capacity of the core scaffold alone. Ferron, despite possessing strong metal-binding potential due to its sulfonic acid group (–SO_3_H) and iodine substituent, is considerably more hydrophilic, which limits its ability to penetrate bacterial membranes and reduces its overall antimicrobial efficacy. These findings demonstrate that, while metal chelation is a necessary prerequisite for activity, the presence of strongly electron-withdrawing groups, such as –NO_2_, combined with favorable physicochemical properties, such as optimal lipophilicity, is critical for maximizing antimicrobial potency [21,22,41,42]. Quinoline-based molecules, quinolin-8-ols, showed potent activity, which may be attributed to their planar aromatic scaffolds and electron-withdrawing substituents (e.g., fluorine or nitro groups). Ciprofloxacin, a fluoroquinolone, contains a carboxyl group and a fluorine at position six on the quinoline ring, both of which enhance DNA gyrase binding and bacterial cell penetration. Nitroxoline, while structurally related, contains a hydroxyl and nitro group on the quinoline ring, conferring effective chelation and moderate antimicrobial activity in anaerobes [6].

Carbadox, a quinoxaline-di-N-oxide derivative, also shares a nitrogen-containing aromatic core and demonstrated mid-range MICs, possibly due to redox cycling and DNA damage [23]. Tetracycline, although structurally distinct, is a polyketide compound with four fused hydrocarbon rings and functional groups capable of chelating divalent metal ions. This facilitates its ability to bind the 30S ribosomal subunit, thereby inhibiting protein synthesis. Its planar conformation and amphipathic properties contribute to strong bacterial cell penetration and explain its consistently high activity against CRC-associated anaerobes in this study [43].

In contrast, quaternary benzylisoquinoline-derived alkaloids, like sanguinarine, showed minimal activity. Structurally, sanguinarine is a bulky, positively benzo[c]phenanthridine alkaloid, with limited flexibility and cell wall penetration. Its activity is further compromised by susceptibility to bacterial efflux, notably via the AcrB pump in *E. coli*, which was shown to reduce MICs upon inhibition [19,41,44]. Berberine chloride, a quaternary protoberberine alkaloid, exhibited relatively weak antimicrobial activity in vitro, which is consistent with previous reports demonstrating its limited intracellular accumulation due to active efflux mechanisms. However, the in vivo efficacy of berberine chloride has been widely documented and is attributed to multiple factors, including synergistic interactions with other bioactive compounds, inhibition of bacterial efflux pumps, and biotransformation by gut microbiota into more active metabolites. Additionally, berberine exerts indirect antimicrobial effects through modulation of host immune responses and disruption of bacterial biofilm formation. These mechanisms are not adequately captured by standard MIC assays, which may underestimate its therapeutic potential [45,46,47,48].

Metal-chelating compounds, such as zinc pyrithione and bismuth subsalicylate, display moderate antibacterial properties, which may result from membrane disruption and metal ion-induced stress. Notably, the coordination of zinc and pyrithione yields complexes with improved solubility and stability, potentially enhancing their antimicrobial profile compared to pyrithione alone. Similarly, vancomycin and metronidazole, despite being structurally distinct, consistently inhibited anaerobes, reflecting their established spectrum of activity [49].

The selective targeting of CRC-associated bacteria while sparing beneficial commensals would represent an important goal for microbiota-directed strategies in CRC prevention. Agents such as metronidazole and tetracycline, which are already used clinically, may offer supportive benefit in CRC risk reduction, particularly in patients exhibiting dysbiotic signatures enriched with *Fusobacterium* or *Bacteroides* species. The tested compounds exhibited inhibitory activity across CRC-associated bacterial species, consistent with previous findings demonstrating that antimicrobial agents do not uniformly affect gut microbiota but instead induce compound-specific dysbiotic signatures [50,51]. In this context, quinoline derivatives, including nitroxoline and related compounds, have been shown to exert antimicrobial effects through mechanisms such as metal chelation and disruption of bacterial homeostasis, rather than broad-spectrum killing [18]. Such activity may justify further investigation of these compounds as candidates for microbiota-directed interventions [52]. Comparing the corresponding $\bar{x}$MICs defined in the current study against CRC-bacteria with their toxicities to some probiotic strains reported in our previous study [20], the antibiotics tetracycline (1.1 vs 19.8 µg/mL) and ciprofloxacin (1.7 vs 26.6 µg/mL) show the widest gaps. Regarding other antibiotics involved, chloramphenicol reverses the relationship and hits probiotics ($\bar{x}$MIC = 6.2 µg/mL) harder than CRC-bacteria ($\bar{x}$MIC = 15.6 µg/mL), whereas ceftriaxone and vancomycin offer rather insignificant advantages against CRC-bacteria ($\bar{x}$MICs = 16.5 and 19 µg/mL, respectively) once probiotics ($\bar{x}$MICs = 23.6 and 47.8 µg/mL, respectively) are considered. Similarly, synthetic analogs of phytochemicals nitroxoline and zinc pyrithione inhibit CRC-pathogens ($\bar{x}$MICs = 10.9 and 16.6 µg/mL, respectively) at concentrations slightly below those affecting probiotics ($\bar{x}$MICs = 20 and 21.3 µg/mL, respectively). Although chloroxine showed weaker effect against CRC-bacteria ($\bar{x}$MICs = 30 µg/mL), the potential selectivity can also be discussed ($\bar{x}$MICs = 245 µg/mL). Oxyquinoline would in principle be highly probiotic-sparing ($\bar{x}$MICs = 743 µg/mL), but its weak activity against CRC-bacteria ($\bar{x}$MICs = 261 µg/mL) makes the selectivity moot. The remaining phytochemicals included in both studies are weak against CRC-bacteria and additionally hit probiotics at lower concentrations, giving no usable selectivity. However, not all compounds tested herein were evaluated for inhibitory activity against probiotic strains in our previous study. Future studies should also assess compound selectivity toward beneficial commensal microorganisms using more complex microbiota-relevant models that go beyond standard in vitro susceptibility assays.

Compounds such as nitroxoline and zinc pyrithione represent candidates for future investigation and warrant further study. Nitroxoline is an approved urinary antiseptic, while zinc pyrithione has historically been used in topical antidandruff formulations; however, its use in EU cosmetic products has been prohibited since 1 March 2022 under Regulation (EU) 2021/1902 (CMR classification). Outside the EU, topical approval for specific indications remains, and its ionophore mechanism, disrupting microbial zinc and copper homeostasis [53], retains pharmacological relevance. Repurposing this would require targeted pharmaceutical formulation for colonic delivery. These features highlight their pharmaceutical repurposing potential [54]. Their potential application as microbiota-modulating agents in the context of CRC prevention warrants further preclinical investigation, particularly in combination with immunotherapy or chemotherapy, where gut microbiota composition has been shown to influence therapeutic response [55].

However, safety considerations are critical. Broad-spectrum antibiotics often lack selective inhibition, disrupting beneficial microbiota and increasing the risk of side effects. This non-selectivity has limited the effectiveness of antibiotic-based preventive strategies in CRC [56]. Sanguinarine and carbadox carry genotoxic or carcinogenic risks that prohibit their use in human medicine. Likewise, poor systemic bioavailability or intestinal absorption limits the application of certain phytochemicals unless targeted formulations are developed [57]. Future work should prioritize compounds demonstrating low cytotoxicity, targeted microbial action, and favorable pharmacokinetics in the colonic environment. Tetracycline, while broadly effective, is known for side effects such as hepatotoxicity and phototoxicity, particularly in children and pregnant women [58]. Ciprofloxacin has been associated with tendinopathy and QT prolongation (it can interfere with cardiac electrical activity), requiring cautious use [59]. In contrast, nitroxoline offers a favorable safety profile, historically used in urinary infections with minimal systemic toxicity [60]. However, carbadox poses significant safety concerns, including genotoxicity and hepatocarcinogenicity in animal models, rendering it unsuitable for human use [61]. Overall, this study identifies several promising compounds for further investigation against CRC-associated bacteria, highlights structure-based predictors of antibacterial activity and supports future research into microbiota-directed strategies for CRC prevention.

### Study Limitations

Several limitations of the present study should be acknowledged. First, all experiments were performed in vitro using planktonic bacterial cultures under controlled laboratory conditions. Consequently, the observed antibacterial activities may not fully reflect the complex interactions occurring within the human intestinal environment, where microbial communities exist in highly dynamic and metabolically interconnected ecosystems. Second, despite previous evaluation of most tested compounds against probiotic strains, the present study was limited to CRC-associated bacterial species and did not include beneficial commensal microorganisms. As a result, conclusions regarding selective microbiota modulation should be considered preliminary and require further experimental validation. Third, bacterial susceptibility was evaluated using MIC determination only. Biofilm formation, microbial interactions, virulence factor expression, and host–microbe interactions were not investigated, despite their recognized importance in colorectal carcinogenesis and bacterial persistence within the intestinal environment. Finally, although the study provides valuable insights into SARs, the analysis was based on a limited set of 18 compounds representing several distinct chemical classes. Therefore, the identified structural trends should be regarded as exploratory observations that require confirmation using larger and more structurally diverse compound libraries. Future studies should incorporate commensal bacterial species, biofilm and co-culture models, targeted colonic delivery approaches, pharmacokinetic evaluation, and in vivo validation to better assess the translational potential of the most active compounds identified in this work.

## 4. Materials and Methods

### 4.1. Chemicals

A range of compounds including phytochemicals (berberine chloride, oxyquinoline, salicylic acid, tannic acid, and sanguinarine chloride), their synthetic analogs (bismuth subsalicylate, chloroxine, nitroxoline, ferron, and zinc pyrithione), and antibiotics (ceftriaxone sodium, ciprofloxacin, chloramphenicol, metronidazole, tetracycline, and vancomycin hydrochloride) were procured from Sigma-Aldrich (Prague, Czech Republic). Dimethyl sulfoxide (DMSO) (Sigma-Aldrich, Prague, Czech Republic) was employed for the preparation of stock solutions for all compounds except metronidazole, salicylic acid, vancomycin hydrochloride, and zinc pyrithione, which were dissolved in distilled water. Stock solutions of chloramphenicol, tannic acid, and tetracycline were prepared using 96% ethanol (Sigma-Aldrich, Prague, Czech Republic).

### 4.2. Microorganisms and Growth Media

The antibacterial activity was assessed against seven strains of Gram-positive/negative and aerobic/anaerobic CRC-associated bacteria sourced from various collections. Standard American Type Culture Collection (ATCC) strains, namely *B. fragilis* ATCC 25285, *C. septicum* ATCC 12464, *E. coli* ATCC 35218, and *S. bovis* ATCC 33317, were obtained from Oxoid [Basingstoke (UK)]. *F. necrophorum* CCM 5981 and *P. anaerobius* CCM 3790 were purchased from The Czech Collection of Microorganisms (CCM) [Brno (Czech Republic)], while *F. nucleatum* CNCTC 5414 (ATCC 10953) was obtained from the National Reference Laboratory—Czech National Collection of Type Cultures [Prague (Czech Republic)]. Brain Heart Infusion (BHI) broth, enriched with 5 g/L soya peptone and 0.5 g/L cysteine, was utilized for most bacteria except for *E. coli* and *C. septicum*, which were cultured in Mueller–Hinton Broth (MHB) and Wilkins–Chalgren Broth (WCB), respectively. Growth media were purchased from Oxoid [Basingstoke, (UK)]. The bacterial strains included in this study were selected because they represent some of the most frequently reported microorganisms associated with CRC-related dysbiosis and carcinogenesis, as well as being available from recognized culture collections and culturable under the standard laboratory conditions used in this study. Specifically, *B. fragilis*, *E. coli*, *F. nucleatum*, *P. anaerobius*, and *S. bovis* have been repeatedly implicated in CRC development through mechanisms involving chronic inflammation, genotoxin production, immune modulation, and promotion of tumor growth. *C. septicum* and *F. necrophorum* were additionally included because of their documented association with CRC and their comparatively limited characterization in antimicrobial susceptibility studies. The selected panel comprises both Gram-positive and Gram-negative species, as well as aerobic and anaerobic bacteria, providing a representative model for evaluating the antibacterial activity of structurally diverse compounds against CRC-associated microorganisms.

### 4.3. Determination of the Minimum Inhibitory Concentration (MIC)

The growth-inhibitory effects of the studied compounds were evaluated by the broth microdilution method using 96-well microtiter plates, following the protocols of CLSI guidelines for aerobic bacteria [62], with slight adjustments tailored for evaluating the anti-infective potential of natural products [63]. The investigation of anaerobic bacteria followed the methodologies outlined by Hecht et al. [64]. Bacteria were sub-cultured in the appropriate media at 37 °C for 24 h, prior to testing. The obligate anaerobes were cultured for 48 h using Whitley A35 Anaerobic Workstation (Don Whitley Scientific, Bingley, UK) where anaerobic conditions were created by the supply of a standard anaerobic gas mixture of 10% H_2_, 10% CO_2_, and 80% N_2_ (Linde Gas, Prague, Czech Republic) at temperature of 37 °C. All compounds were diluted two-fold (concentrations ranging from 512 to 1 μg/mL) and standard antibiotics (with starting concentration of 32 μg/mL) in appropriate growth media using the Freedom EVO 100 automated pipetting platform (Tecan, Mannedorf, Switzerland) or multichannel pipette (Eppendorf, Hamburg, Germany). Bacterial inocula were adjusted to the 0.5 McFarland standard solely by spectrophotometric measurement using a Densi-La-Meter II (Lachema, Brno, Czech Republic), corresponding to approximately 1.5 × 10^8^ CFU/mL. Plates were inoculated by bacterial suspension (5 μL/well) and incubated for 24 h at 37 °C in a Biological Thermostat (Memmert GmbH & Co. KG, Buchenbach, Germany) whereas the plates with anaerobes were handled under anaerobic conditions in the Whitley A35 Anaerobic Workstation (Don Whitley Scientific, Bingley, UK). Prior to optical density measurement, each well was visually inspected for the presence or absence of bacterial growth. Although CLSI guidelines define MIC as the lowest concentration preventing visible growth, determination of antimicrobial activity for phytochemicals and related compounds may be complicated by trailing growth endpoints. Therefore, consistent with previously published studies on natural products [55,65], the optical density of the cultures was measured using a Cytation 3 Imaging Reader (BioTek, Winooski, VT, USA) before and after growth at 405 nm. The lowest concentration (μg/mL) of test compounds, at which the bacterial growth was inhibited by ≥80%, was defined as MIC [66,67]. The samples were assayed using multiplate design and all tests were performed as three independent experiments, each carried out in triplicate. According to the widely accepted norm in MIC testing, the mode and median were used for the final value calculation when the triplicate endpoints were within the two- and three-dilution range, respectively. Control experiments performed in the absence of test compounds confirmed that DMSO and 96% ethanol had no inhibitory effect on the growth of any tested bacterial strain at the assayed final concentrations, which did not exceed 1% (*v*/*v*).

### 4.4. Statistical Analysis

The MIC values were analyzed using a linear mixed-effects model to evaluate whether antimicrobial activity differed among compound classes and bacterial species. Compound class, bacterium, and their interaction were included as fixed effects, while compound identity was included as a random intercept to account for repeated measurements of the same compounds against different bacteria. The fitted model was MIC~structural group × bacterium + (1|compound). The compound classes were antibiotics, quinolines, quinoxaline-di-N-oxides quaternary benzylisoquinoline-derived alkaloids, (poly)phenols, and pyrithione complexes. For the calculation of $\bar{x}$MICs, values greater than the maximum tested concentration, 32 µg/mL for antibiotics and 512 µg/mL for phytochemicals and synthetic analogs, were replaced by 64 and 1024 µg/mL. The analysis was performed in Jamovi using the GAMLj mixed-model module [68].

To explore relationships among the tested compounds based on their antibacterial activity profiles, hierarchical clustering and heatmap analysis were performed using MIC values obtained against all seven CRC-associated bacterial strains. MIC values were log_2_-transformed prior to analysis to reduce data skewness and facilitate comparison across compounds. Values exceeding the maximum tested concentration (32 µg/mL for antibiotics and 512 µg/mL for phytochemicals and synthetic analogs) were replaced by 64 and 1024 µg/mL, respectively, prior to analysis. Hierarchical clustering was conducted using Euclidean distance as the similarity metric and Ward’s linkage method for cluster aggregation. The resulting dendrogram and heatmap were generated to visualize similarities in antibacterial activity patterns among the tested compounds and to identify potential relationships between chemical structure and biological activity. Heatmap generation and hierarchical clustering were performed in Jamovi version 2.6 [68].

## 5. Conclusions

This study presents a comprehensive evaluation of the in vitro antibacterial activity of phytochemicals, synthetic analogs, and antibiotics against CRC-associated bacterial strains, with a focus on their SAR. Among the 18 tested compounds, conventional antibiotics, particularly tetracycline and ciprofloxacin, demonstrated the broadest and most potent inhibitory effects. Moderate activity was also observed for several synthetic compounds, such as nitroxoline, carbadox, and zinc pyrithione, which showed promise as candidates for further evaluation. In contrast, quaternary benzylisoquinoline-derived alkaloids and polyphenols exhibited poor efficacy, likely due to unfavorable physicochemical properties. Novel MIC data were reported for ferron, oxyquinoline, and chloroxine against the majority of tested CRC-associated strains, filling an important gap in the literature. SAR analysis suggests that the 8-hydroxyquinoline scaffold, particularly when combined with a C-5 electron-withdrawing substituent, as exemplified by nitroxoline, may contribute to enhanced antibacterial activity within the quinoline class. Quaternary benzylisoquinoline-derived alkaloids (berberine, sanguinarine chlorides) and polyphenols showed consistently poor in vitro activity, attributable to efflux-mediated resistance and physicochemical limitations rather than absence of pharmacological potential. Compounds containing aromatic heterocyclic scaffolds, electron-withdrawing substituents, and metal-chelating functionalities generally exhibited enhanced growth-inhibitory activity, highlighting the importance of molecular properties in determining antibacterial performance. However, the observed activity patterns were established under in vitro conditions and may be influenced by additional factors present in the intestinal environment, including microbial interactions, biofilm formation, local pH, metabolism, and compound bioavailability. Therefore, the identified SARs should be regarded as preliminary observations that provide a basis for future investigations aimed at understanding how physicochemical properties influence antimicrobial activity within complex gut microbial ecosystems. Future studies should investigate the bactericidal vs. bacteriostatic nature of active compounds (MBC determination), their activity under sub-MIC conditions relevant to virulence factor expression (BFT, FadA), and their pharmacokinetic behavior in targeted colonic delivery formulations. Additionally, it should be investigated whether these compounds selectively inhibit CRC-associated bacteria while sparing indigenous beneficial gut microbiota, particularly strains with potential oncopreventive functions.

## Figures and Tables

**Table 1:** In vitro growth-inhibitory effects of phytochemicals, their synthetic analogs, and antibiotics against bacterial strains causing colorectal cancer.

Compound	Class	Bacterium/Minimum Inhibitory Concentrations [μg/mL]							$\bar{x}$MIC ± SD [μg/mL]
		*B. fragilis*	*C. septicum*	*E. coli*	*F. necrophorum*	*F. nucleatum*	*P. anaerobius*	*S. bovis*	
Tetracycline	A	0.5	0.125	2	1	1	2	1	1.1 ± 0.7
Ciprofloxacin	A	8	0.5	0.0625	0.5	0.5	0.5	2	1.7 ± 2.8
Nitroxoline	Q	4	8	8	16	16	16	8	10.9 ± 5.0
Carbadox	QX	4	4	32	0.5	4	0.5	32	11.0 ± 14.4
Chloramphenicol	A	8	8	>32	8	16	1	4	15.6 ± 21.8
Ceftriaxone	A	>32	8	0.25	32	2	8	1	16.5 ± 23.6
Zinc pyrithione	PC	8	16	4	16	32	32	8	16.6 ± 11.4
Metronidazole	A	0.5	>32	2	0.25	0.125	1	>32	18.8 ± 30.9
Vancomycin	A	32	8	>32	4	16	1	8	19.0 ± 22.3
Chloroxine	Q	32	32	16	32	32	>32	4	30.3 ± 18.5
Sanguinarine chloride	qBIAs	32	32	>32	32	>32	>32	16	43.4 ± 20.1
Olaquindox	QX	>32	>32	32	32	>32	16	512	112.0 ± 177.4
Oxyquinoline	Q	32	128	128	512	512	512	4	261.1 ± 239.1
Ferron	Q	128	256	512	512	512	512	256	384.0 ± 165.2
Salicylic acid	PP	128	>512	>512	>512	>512	>512	128	768.0 ± 4437.2
Bismuth subsalicylate	PP	32	>512	>512	>512	>512	>512	>512	882.3 ± 374.9
Berberine chloride	qBIAs	>512	>512	>512	>512	>512	>512	256	914.3 ± 290.3
Tannic acid	PP	>512	>512	>512	>512	>512	>512	512	950.9 ± 193.5

Class: Quinolines (Q), quinoxaline-di-N-oxides (QX), quaternary benzylisoquinoline-derived alkaloids (qBIAs), (poly)phenols (PP), pyrithione complex (PC), antibiotics (A). $\bar{x}$MIC = arithmetic mean minimum inhibitory concentration, calculated from MIC values obtained against all seven tested bacterial strains. For $\bar{x}$MIC calculation, values exceeding the maximum tested concentration (32 µg/mL for antibiotics and 512 µg/mL for phytochemicals and synthetic analogs) were replaced by 64 and 1024 µg/mL, respectively. SD = standard deviation.

**Table 2:** Structural classes with the lowest estimated marginal mean MICs (µg/mL) for each CRC-causing bacterium.

Bacterium	Lowest Class Overall	Estimated Marginal Mean MIC (µg/mL)	Lowest Non-Antibiotic Class	Estimated Marginal Mean MIC (µg/mL)
*B. fragilis*	PC	8	PC	8
*C. septicum*	A	14.77	PC	16
*E. coli*	PC	4	PC	4
*F. necrophorum*	A	7.63	PC/QX	16.00/16.25
*F. nucleatum*	A	5.94	PC	32
*P. anaerobius*	A	2.25	QX	8.25
*S. bovis*	PC	8	PC	8

Class: Antibiotics—A, quinoxaline-di-N-oxides—QX, pyrithione complex—PC.

## Data Availability

The data supporting the findings of this study are available within the article. No additional datasets were generated or deposited in external repositories.

## Abbreviations

The following abbreviations are used in this manuscript:

A	Antibiotics
ATCC	American Type Culture Collection
BHI	Brain Heart Infusion
CCM	Czech Collection of Microorganisms
CFU	Colony-Forming Unit
CLSI	Clinical and Laboratory Standards Institute
CNCTC	Czech National Collection of Type Cultures
CRC	Colorectal Cancer
DMSO	Dimethyl Sulfoxide
qBIAs	Quaternary Benzylisoquinoline-Derived Alkaloids
MHB	Mueller–Hinton Broth
MIC	Minimum Inhibitory Concentration
PC	Pyrithione Complex
PP	(Poly)phenols
Q	Quinolines
QX	Quinoxaline-di-*N*-Oxides
WCB	Wilkins–Chalgren Broth
$\bar{x}$MIC	Arithmetic Mean MIC
